# Chinese Herbal Preparation Xuebijing Potently Inhibits Inflammasome Activation in Hepatocytes and Ameliorates Mouse Liver Ischemia-Reperfusion Injury

**DOI:** 10.1371/journal.pone.0131436

**Published:** 2015-07-01

**Authors:** Xiqiang Liu, Zhiqiu Hu, Bin Zhou, Xiang Li, Ran Tao

**Affiliations:** 1 Department of Surgery, Ruijin Hospital, Shanghai Jiaotong University School of Medicine, Shanghai, PR China; 2 Department of Surgery, Minhang Hospital, Fudan University, Shanghai, PR China; 3 Department of cardiothoracic surgery, Zhejiang Provincial People’s Hospital (ZJPPH), Hangzhou, Zhejiang Province, PR China; 4 Department of Pharmacology, Faculty of Basic Medicine, Zhejiang Medical College, Hangzhou, Zhejiang Province, PR China; 5 Department of Hepatobiliary-Pancreatic Surgery, Zhejiang Provincial People’s Hospital (ZJPPH), Hangzhou, Zhejiang Province, PR China; National Institutes of Health, UNITED STATES

## Abstract

The Chinese herb preparation Xuebijing injection (XBJ) has been widely used in the management of various septic disorders or inflammation-related conditions, however the molecular mechanism of its anti-inflammatory effect remains largely elusive. In the current study, we found that XBJ treatment potently ameliorated mouse hepatic ischemia-reperfusion (IR) injury, manifested as decreased liver function tests (LDH, ALT, AST), improved inflammation and less hepatocyte apoptosis. Notably, XBJ markedly inhibited inflammasome activation and IL-1 production in mouse livers subjected to IRI, even in the absence of Kupffer cells, suggesting Kupffer cells are not necessary for hepatic inflammasome activation upon Redox-induced sterile inflammation. This finding led us to investigate the role of XBJ on hepatocyte apoptosis and inflammasome activation using an in vitro hydrogen peroxide (H_2_O_2_)-triggered hepatocyte injury model. Our data clearly demonstrated that XBJ potently inhibited apoptosis, as well as caspase-1 cleavage and IL-1β production in a time- and dose-dependent manner in isolated hepatocytes, suggesting that in addition to its known modulatory effect on NF-κB-dependent inflammatory gene expression, it also has a direct impact on hepatocyte inflammasome activation. The current study not only deepens our understanding of how XBJ ameliorates inflammation and apoptosis, but also has immediate practical significance in many clinical situations such as partial hepatectomy, liver transplantation, etc.

## Introduction

Liver ischemia-reperfusion injury (IRI) is a process by which an hypoxic insult and subsequent re-establishment of blood flow leads to exogenous, antigen-independent inflammation[1]. The sequence of events is characterized by the generation of reactive oxygen species (ROS) and further propagation of liver dysfunction and damage resulting from secondary sterile inflammatory response[2,3]. To date, antioxidant therapy has been proven useful[4]. Additionally, the inflammatory cascade can be suppressed with various biochemical treatment to ameliorate the recruitment of ROS-producing leukocytes[5–7]

XBJ is derived from a mixture of traditional Chinese herbs including Flos Carthami, Radix Paeoniae Rubra, Rhizoma Chuanxiong, Radix Salviae Miltiorrhizae and Radix Angelicae Sinensis[8]. It has been extensively used in the clinical management of severe sepsis, with dramatically decreased secretion of TNF-α, IL-6, and IL-8 as well as significantly improved patient survival[9,10]. A recent study using bioactivity-integrated ultra-performance liquid chromatography/quadrupole time-of-flight mass spectrometry (UPLC-Q/TOF) assay system identified 9 potential anti-inflammatory ingredients, including gallic acid, danshensu, protocatechualdehyde, hydroxysafflor yellow A (HSYA), oxypaeoniflorin, paeoniflorin, safflor yellow A, senkyunolide I and benzoylpaeoniflorin, as NF-κB inhibitors[11]. Among all these components, HSYA is probably the most well studied substance in the management of many inflammation related entities as well as innate immunity elicited by various exogenous or endogenous danger signals. For example, XBJ and HSYA have been proved to be effective in several experimental models of lung injury. In sepsis-related lung injury, XBJ potently ameliorated lung vascular permeability and inflammatory cytokine production via upregulating Toll-interacting protein (Tollip) expression and dampening the activation of toll-like receptor 4 (TLR4) and mitogen-activated protein kinase (MAPK) pathways[12–14]. Similarly, in acute lung inflammatory responses elicited by oleic acid, bleomycin or paraquat, XBJ effectively protected lung injury by inhibiting IL-6 production and promoting IL-10 expression[15], boosted cAMP/PKA pathway activation[16], and inhibiting the activation of the nuclear factor (NF)-κB and p38 MAPK[17,18]. XBJ has also been reported to exert neuroprotective effects. In both rat ischemic stroke and cerebral ischemia-reperfusion models, XBJ ameliorated brain cell apoptosis and increased autophagy via the PI3K/Akt/GSK3β signaling pathway[19,20]. Moreover, in rodent models of Beta-amyloid (Aβ)1-42 or 6-hydroxydopamine (6-OHDA)-induced Parkinson's disease (PD), HSYA effectively protected neurons from apoptosis[21] and inhibited brain inflammation via the JAK2/STAT3/NF-κB pathway[22]. Similarly, HSYA could convey protection to H9c2 cardiomyocytes against anoxia/reoxygenation (A/R)-induced apoptosis through PI3K/Akt/Nrf2-dependent upregulation of heme oxygenase-1 (HO-1) expression[23]. XBJ also improved the survival rate of irradiated mice and attenuated the effects of radiation on hematopoietic injury by decreasing ROS production in bone marrow cells[24].

Many studies also focused on the effect of XBJ in liver-related conditions, especially hepatic fibrosis. HSYA significantly reduced liver fibrosis via downregulation of α-smooth muscle actin (SMA), collagen α type I, matrix metalloproteinases (MMP)-9, and tissue inhibitors of metalloproteinases (TIMP)-1, associated with decreased expression of transforming growth factor (TGF)-β1 and phosphorylation of Smad4[25], and upregulation of the expression of peroxisome proliferator-activated receptor-γ (PPAR-γ) and matrix metallopeptidases-2 (MMP-2)[26,27]. Further in vitro studies revealed that HSYA significantly induced apoptosis of culture-activated hepatic stellate cells (HSCs) in a dose- and time-dependent manner, possibly via ERK1/2 and ERK1/2-regulated gene expression, including Bcl-2, Cytochrome c, caspase-9, and caspase-3[28]. It also suppressed HSC activation by ERK5-mediated myocyte enhancer factor 2 C (MEF2C) down-regulation[29].

The anti-inflammatory effects of XBJ on macrophage and Kupffer cells have been well documented in previous studies. XBJ was able to suppress the inflammatory responses in microglia induced by oxygen glucose deprivation[30]. More recently, HSYA was reported to reduce IR-induced acute liver injury by directly attenuating macrophage activation by down-regulated the expression of MMP-9 and ROS, and inhibited NF-κB activation and P38 phosphorylation under inflammatory conditions[31]. However, whether XBJ has a direct effect on hepatocytes remains obscure.

In the current study, we used both in vivo IRI model and in vitro hydrogen peroxide (H_2_O_2_)-triggered hepatocyte injury model to study the effect of XBJ on hepatic function and attempted to explore the molecular mechanism involved. In particular, we will focus on the inflammasome activation induced by IRI in hepatocytes by depletion of Kupffer cells in vivo in order to evaluate the relative contribution of hepatocytes in liver IRI. Then we try to investigate the effect and mechanism of XBJ on isolated hepatocytes subjected to oxygen free radical induced injury and inflammation, with special attention paid to inflammasome activation.

## Materials and Methods

### Ethics Statement

All animal care and experimental procedures complied with the criteria outlined in the National Guideline for the Care and Use of Laboratory Animals, formulated by the Ministry of Science and Technology of the People’s Republic of China. The animal protocol was approved by the Ethical Committee on Animal Experiments at Ruijin Hospital. All reasonable steps to prevent animal suffering were undertaken. Sacrifice was performed during deep anesthesia.

### Animals

Male wild-type C57BL/6 mice were purchased from the Shanghai Laboratory Animal Center (Chinese Academy of Sciences, Shanghai, China) and used at 8–10 wks of age. The total number of animals used was 84. The animals were housed under specific pathogen-free conditions and had access to food and water ad libum.

### Reagents

A commercially available preparation of XBJ was purchased from Tianjin Hongri Pharmaceutical Stock Co., Ltd. (Tianjin, China) and was used in both in vitro and in vivo studies. The following antibodies were used in the western blot: NACHT domain, leucine-rich repeat [LRR] domain, and pyrin domain [PYD]-containing protein-3 (NLRP3) (1:1000, R&D Systems, Inc., Minneapolis, MN), caspase-1 (1:1000, Abcam, Inc., Cambridge, MA), apoptosis-associated speck-like protein containing a caspase-recruitment domain (ASC) (1:200, Santa Cruz Biotechnology, Inc., Santa Cruz, CA), interleukin-1β (IL-1β) (1:1000, Biovision, Inc., Milpitas, CA), cleaved-caspase-3, B-cell lymphoma 2 (BCL-2), B-cell lymphoma-extra large (BCL-XL) and glyceraldehyde-3-phosphate dehydrogenase (GAPDH) (1:1000 Cell Signaling Technology, Inc., Danvers, MA).

### Liver IR model and treatment

We adopted a mouse model of partial warm hepatic IR in which 70% of the liver mass underwent 90 minutes of warm ischemia followed by 6 hours of reperfusion, as described previously [6,32]. In brief, mice were anesthetized, injected with phenobarbital sodium (60mg/kg, intraperitoneally and heparin (100U/kg, intraperitoneally). Sham-operated mice underwent the same procedure but without vascular occlusion. The IR group were infused with a single dose of XBJ (2ml/kg) or normal saline via the dorsal penile vein. XBJ was administrated either 10 mins prior to occlusion of the arterial and portal venous blood supplying the cephalad lobes (before group) or immediately after lobetic reperfusion (after group), while the control group was treated with the same volume of normal saline and subjected to 90 mins of partial hepatic ischemia. Mice were sacrificed after 6 hours of reperfusion, liver and serum samples were collected for further analysis.

### Determination of ROS level

Hepatic reactive oxygen species (ROS) level was measured using DCFH-DA as a probe. ROS levels were measured using commercially available kits (jiancheng, tianjin, China) according to the manufacturer’s instructions.

### Assessment of hepatocellular damage

Alanine transaminase (ALT), aspartate aminotransferase (AST) and lactate dehydrogenase (LDH) levels were measured using commercially available kits (jiancheng, tianjin, China) according to the manufacturer’s instructions.

### Depletion of Kupffer cells

Mice were intravenously injected with 200μl of clodronate-containing liposomes (lip-clod) or PBS-containing liposomes (lip-PBS, qianyeda, beijing, China). Two days later, Sham or liver IR surgery were performed on these mice

### Isolation and culture of hepatocytes

Primary hepatocytes were isolated from normal C57BL/6 mice as described previously [33–35]. In brief, the portal vein was cannulated and then the vena cava was cut as the outlet. The liver was perfused through the portal vein with perfusion buffer solution (1×PBS with 10mM HEPES[Sigma], 0.05% Kcl[Sigma], 5mM glucose[Sigma], 200uM EDTA[Sigma] and 0.001% phenol red solution[Sigma], PH adjust to 7.4 with 2M NaOH[Sigma]) until the liver became clear of blood. When the liver began to get congested, the liver was continuously perfused with digestive medium (1×PBS with 30mM HEPES, 0.05% Kcl, 5mM glucose, 1mM CaCl_2_[Sigma], 0.02% Collagenase H [Roche] and 0.2% phenol red solution, PH adjust to 7.4 with 2M NaOH) at a flow rate of 7 ml/min for 5 min. When perfusion is complete, the whole liver was dissect out and fractionized with a cell scraper in a petri dish in the presence of Williams E medium (Gibco) with 10% fetal bovine serum (Gibco). Non-parenchymal cells (NPCs) were separated from the hepatocytes by two cycle of differential centrifugation (400 rpm for 2 minutes). Hepatocyte suspensions were separated by 40% percoll centrifugation at 400 rpm for 10 minutes and then washed by centrifugation at 400 rpm for 2 minutes by three times. Hepatocyte purity was constantly more than 98%, as assessed by light microscopy, and cell viability was typically 95% as determined by trypan blue exclusion assay. The cells were maintained for additional 24 hours before treatments.

### Quantitative RT-PCR

Quantitative polymerase chain reaction was performed using the DNA Engine with the Chromo 4 detector (MJ Research, Waltham, MA). 1× SuperMix (Platinum SYBR Green quantitative polymerase chain reaction kit, Invitrogen, Carlsbad, CA), complementary DNA, and each primer pair were added to the reaction tube to reach a final volume of 20μl. Target gene expressions were calculated by their ratios to the housekeeping gene, hypoxanthine-guanine phosphoribosyl transferase (HPRT). The sequence of primer sets used for target genes could be found in Table 1.

**Table 1:** Sequence of primer pairs used in real-time quantitative PCR.

Genes	Forward primer	Reverse primer
IL-6	CCAATGCTCTCCTAACAGA	TGTCCACAAACTGATATGC
TNF-α	CTCTTCAAGGGACAAGGCTG	CTCTTCAAGGGACAAGGCTG
IL-1β	TTCAGGCAGGCAGTATCA	GTCACACACCAGCAGGTTA
CXCL-1	GGGAGGCTGTGTTTGTATG	AATGTCCAAGGGAAGCGTC
CXCL-10	CCAAGTGCTGCCGTCATTTTC	GGCTCGCAGGGATGATTTCAA
HPRT	TCAACGGGGGACATAAAAGT	TGCATTGTTTTACCAGTGTCAA

### Western Blot

Tissue or cell samples were prepared in lysis buffer containing 1% Triton X-100 and a mixture of protease inhibitors, and quantified using Pierce BCA protein assay kit (Thermo scientific, Rockford, IL). Proteins from supernatants were isolated by precipitation. 500 μl of supernatant was mixed with 500 μl of methanol and 150 μl of chloroform, respectively, and centrifuged at 14,000 rpm for 10 min. The aqueous phase was discarded and the protein pellet was washed with 800 ul methanol to dry the pellet and added with 20–50 μl lysis buffer. Equal amounts of protein samples were separated by SDS-PAGE on an 8–15% bis-tris gel and electrophoretically transferred onto 0.2 μm polyvinylidene fluoride membrane (Merck Millipore, Merck KGaA, Darmstadt, Germany). Membranes were blocked with 5% non-fat milk and sequentially incubated with primary and HRP-conjugated secondary antibodies. Bound antibodies were visualized using enhanced chemiluminescent substrate (ECL; thermo scientific, Waltham, MA).

### ELISA

Serum levels of monocyte chemotactic protein 1 (MCP-1), interleukin-6 (IL-6), tumor necrosis factor-α (TNF-α) and interleukin-1β (IL-1β) in supernatant of primary hepatocyte cultures were measured using mouse ELISA kits (eBioscience, San Diego, CA) according to the protocol provided by the manufacturer. A detection antibody and Avidin-HRP were used to detect IL-1β, IL-6, MCP-1 and TNF-α protein signals. The plates were read at 450 nm using a Microplate Reader Model 450 (Bio-Rad, Hercules, CA). All experiments were performed at least three times in triplicate.

### Histology and immunohistochemistry

Perfused livers were fixed with 4% paraformaldehyde for 24 hours and then embedded in paraffin. For detection of liver necrosis and apoptosis, formalin-fixed liver tissue sections were stained with hematoxylin and eosin (H&E) for evaluation of necrosis. For apoptosis detection, tissue sections were stained with terminal deoxynucleotidyl transferase (TdT)-mediated dUTP digoxigenin nick-end labeling (TUNEL) according to the manufacturer’s instructions (In situ cell death detection kit, POD, Roche). For immunohistochemical detection of infiltrating inflammatory cells, primary mAbs against mouse Ly-6G (1A8; BD Biosciences, San Jose, CA) and CD68 (FA-11; AbD Serotec, Raleigh, NC) were used on liver specimens, and positive cells were counted in 15 high power fields (HPF)/section for quantification of infiltrating immune cells.

### Myeloperoxidase (MPO) assay

The hepatic MPO activity were measured using the commercially available myeloperoxidase peroxidation fluorometric assay kits (Cayman Chemical Company, Ann Arbor, MI) according to the protocol provided by the manufacturer. One absorbance unit (U) of MPO activity was defined as the quantity of enzyme degrading 1 mol peroxide/min at 25°C/gram of tissue.

### Network pharmacology technology

The basic gene list associated with inflammation was collected from the HuGE Navigator (version 2.0)[36]. Human Protein Reference Database (HPRD) curated human protein-protein interaction (PPI)[37] was applied to construct inflammation associated network according to the methods we used previously to construct Coronary Heart Disease (CHD) database[38]. Cytoscape 3.1.0 was applied to analyze and visualize the network[39]. The targets of XBJ were highlighted with red color, which were carried out with experimental validation through different levels.

### Statistics

Statistical analysis was performed using the Student t test or one-way ANOVA. All statistical analyses were performed using GraphPad Prism 5. Graphs were also generated using GraphPad Prism 5. The p value < 0.05 was considered statistically significant.

## Results

### 1. XBJ significantly attenuated mouse liver IRI

**Fig 1:**
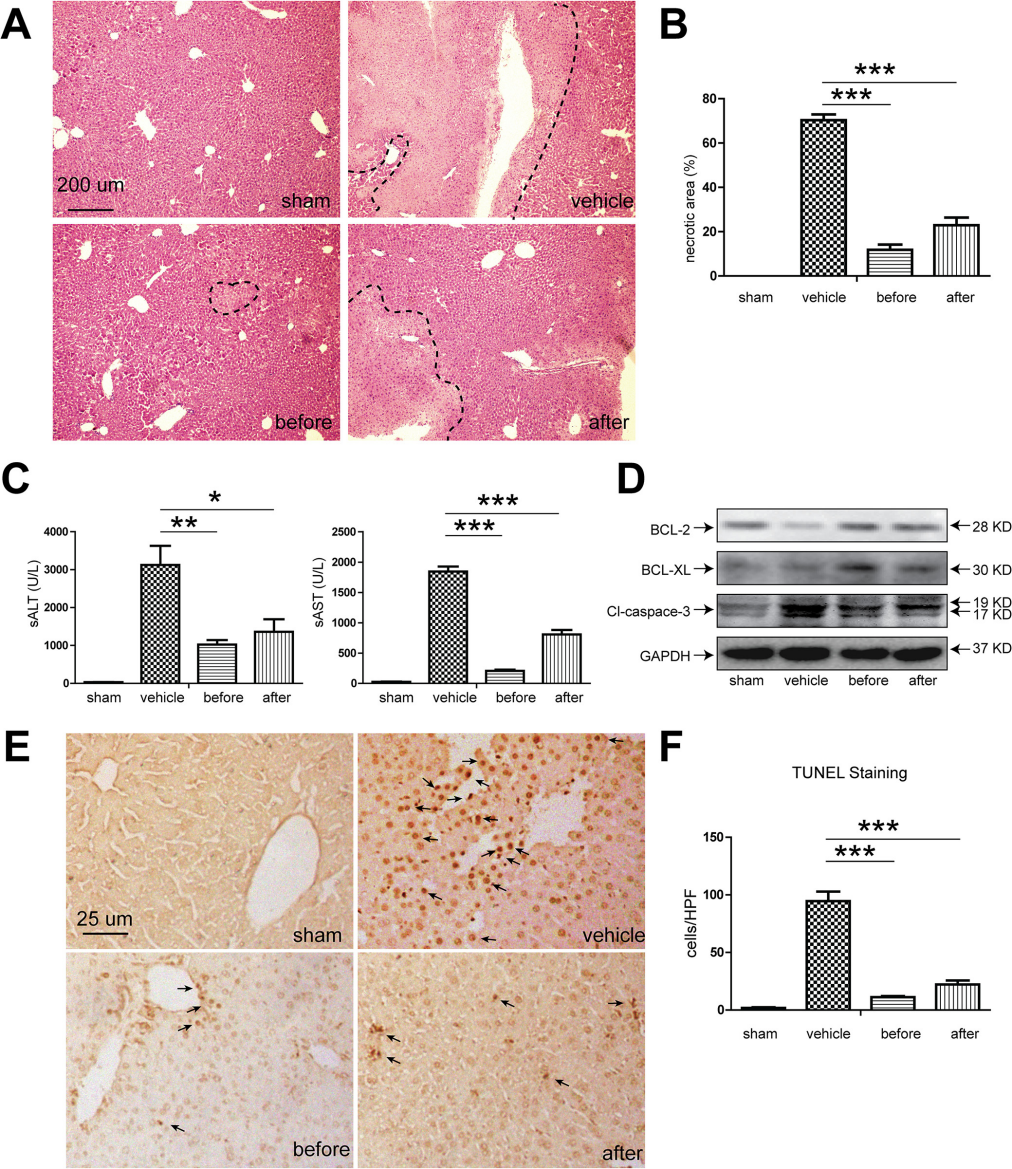
XBJ treatment ameliorated hepatic injury and apoptosis. B6 mice were either sham operated (sham group) or subjected to 90 minutes of partial warm ischemia, followed by 6 hours of reperfusion. XBJ (2 ml/kg iv) was given either before ischemia (before group) or after initiation of reperfusion (after group), while the same volume of normal saline was administered in the vehicle group. Serum and liver samples were harvested (n = 3-6/group). Liver damage was analyzed by (A and B) liver histology (representative H&E staining; Original magnifications, 100×magnification) and (C) serum ALT and AST levels. (D) Western blot analysis of BCL-2, BCL-XL and cleaved-caspase-3 in IR liver lobes. Representative blots from at least three mice were shown. (E) Detection of apoptotic cells (dark arrows) within the IR hepatic lobes with TUNEL staining (Original magnifications, 400×magnification). (F) Quantification of TUNEL positive cells in IR hepatic lobes with or without XBJ treatment. TUNEL positive cell were calculated within 15 randomly selected fields. Data were expressed as mean±SEM. *P<0.05, **P<0.01, *** P <0.001.

We started by looking at the effect of XBJ on a well-established mouse partial non-lethal IR model. XBJ was given either before ischemia or after initiation of reperfusion in the same volume of normal saline that was administrated in the vehicle group. We found that XBJ significantly attenuated hepatocyte necrosis, with better preserved microscopic structures (Figs 1A and 5B). The serum levels of AST and ALT were significantly reduced after XBJ treatment, especially in the before ischemia group, indicating less hepatocyte injury (Fig 1C). Since apoptosis represents a major mechanism in hepatic IR, we also looked at the hepatocyte apoptosis with or without XBJ treatment. XBJ treatment significantly decreased the Caspase-3 cleavage, with increased expression of the anti-apoptotic proteins BCL-2 and BCL-XL, suggesting that the hepatocyte apoptosis in IR liver tissues was significantly reduced upon XBJ treatment (Fig 1D). Along with this line, TUNEL staining also revealed much less intra-hepatic apoptotic bodies (Fig 1E and 1F).

### 2. XBJ significantly ameliorated inflammation within mouse livers that have undergone IR

**Fig 2:**
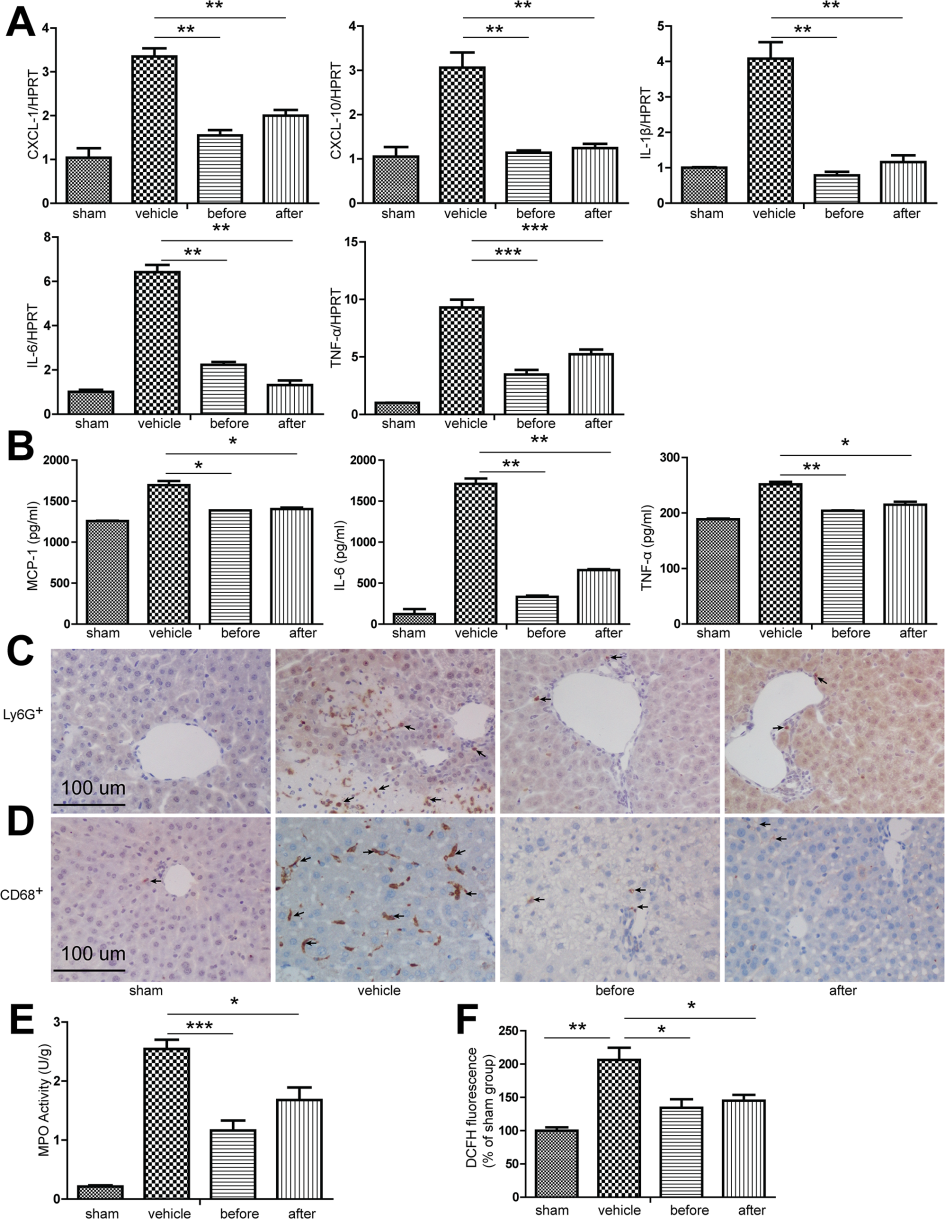
XBJ significantly ameliorated inflammation in mouse livers that have undergone IRI. B6 mice were either sham operated (sham group) or subjected to 90 minutes of partial warm ischemia, followed by 6 hours of reperfusion. XBJ (2 ml/kg iv) was given either before ischemia (before group) or after initiation of reperfusion (after group), while the same volume of normal saline was administered in the vehicle group. Serum and liver samples were harvested (n = 3-6/group). (A) qRT-PCR detection of CXCL-1, CXCL-10, IL-1β, IL-6 and TNF-a. Data were normalized to HPRT gene expression and expressed as fold increase above the sham group (set as 1). (B) ELISA detection of MCP-1, IL-6 and TNF-α. Accumulation of neutrophils and macrophages after administration of XBJ was analyzed by (C) Ly-6G (neutrophils), (D) CD68 (macrophages) immunohistochemical staining (Original magnifications, 200×magnification) and (E) MPO levels in IRI liver lobes. (F) ROS was detected using the fluorescent probe DCFH-DA. Data were expressed as mean±SEM. *P<0.05, **P<0.01,*** P <0.001.

**Fig 3:**
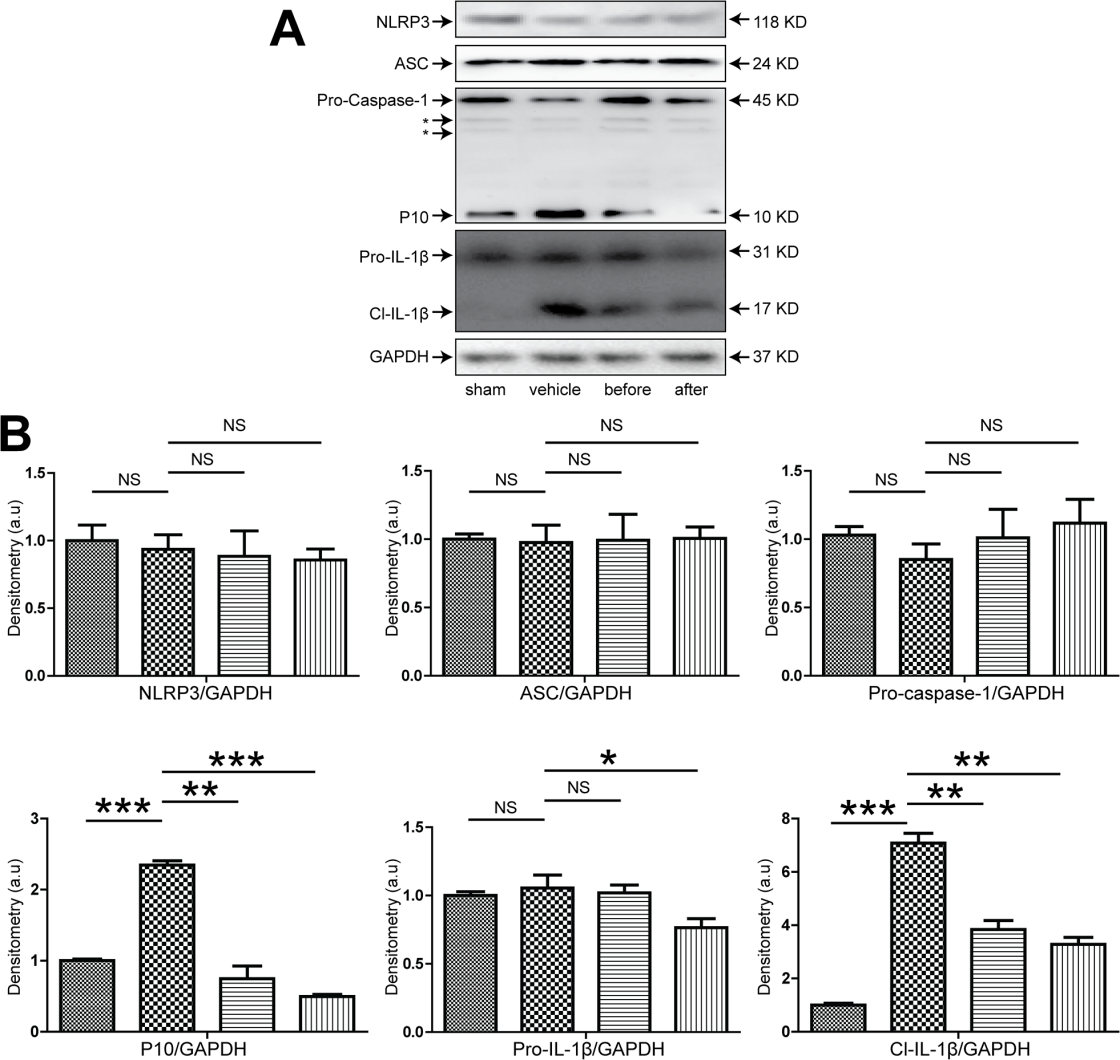
XBJ suppressed inflammasome activation in mouse livers that have undergone IRI. B6 mice were either sham operated (sham group) or subjected to 90 minutes of partial warm ischemia, followed by 6 hours of reperfusion. XBJ (2 ml/kg iv) was given either before ischemia (before group) or after initiation of reperfusion (after group), while the same volume of normal saline was administered in the vehicle group. Liver samples were then harvested (n = 3-6/group). (A) Western blot analysis of NLRP3, ASC, Caspase-1 and IL-1β in IRI liver lobes. Representative blots from at least three mice were shown. *, non-specific bands. (B) Densitometric analyses of western blot for NLRP3, ASC, Caspase-1 and IL-1β. Values shown were relative to sham group (set as 1). Compared to sham group, *p<0.05; **p<0.01; ***p<0.001. NS, no significance.

IR elicited robust innate immunity within the liver tissues, so we checked the cytokine profile within the IR lobes as well as serum levels of several pro-inflammatory cytokines/chemokines. A previous study has shown that XBJ exerted its effects via inhibition of the NF-κB-dependent transcription of several pro-inflammatory genes, such IL‑6 and TLR4 [12,15]. Consistent with this effects, we found that XBJ treatment either before partial warm ischemia or after reperfusion dramatically decreased the intra-hepatic expression of cytokine IL-1β, IL-6, TNF-α as well as the chemokines C-X-C motif ligand (CXCL)1 and CXCL10 (Fig 2A). The serum levels of MCP-1, IL-6 and TNF-α were also dramatically decreased (Fig 2B). To further prove that the intra-hepatic infiltration of pro-inflammatory cells were decreased after use of XBJ, we used immunohistochemical staining and found the intra-hepatic expression of LY6G which represented neutrophils and CD68 which represented macrophages were also significantly decreased (Fig 2C and 2D). the MPO activity within the IR liver lobes was dramatically decreased after use of XBJ (Fig 2E). Lastly, Hepatic ROS level was measured using DCFH-DA as a probe. The hepatic level of DCFH fluorescence was significantly increased after IR, and the increase of hepatic DCFH fluorescence was prevented in the IR liver lobes treated with XBJ (Fig 2F). Given IL-1β is mainly generated through inflammasome activation, we next examined whether XBJ treatment affected the inflammasome related protein expression. XBJ treatment did not significantly alter the expression of NLRP3 and ASC per se, two main components of the inflammasome protein complex. However, the cleavage of pro-Caspase-1 and pro-IL-1β which was known to be the key step in the processing of mature IL-1β within the inflammasome was significantly decreased (Fig 3). These data clearly indicated that XBJ attenuate liver IRI by minimizing both hepatocyte apoptosis as well as inflammasome activation within the IR liver.

### 3. Therapeutic effect of XBJ on hepatic IRI and inflammasome activation in the absence of Kupffer cells

**Fig 4:**
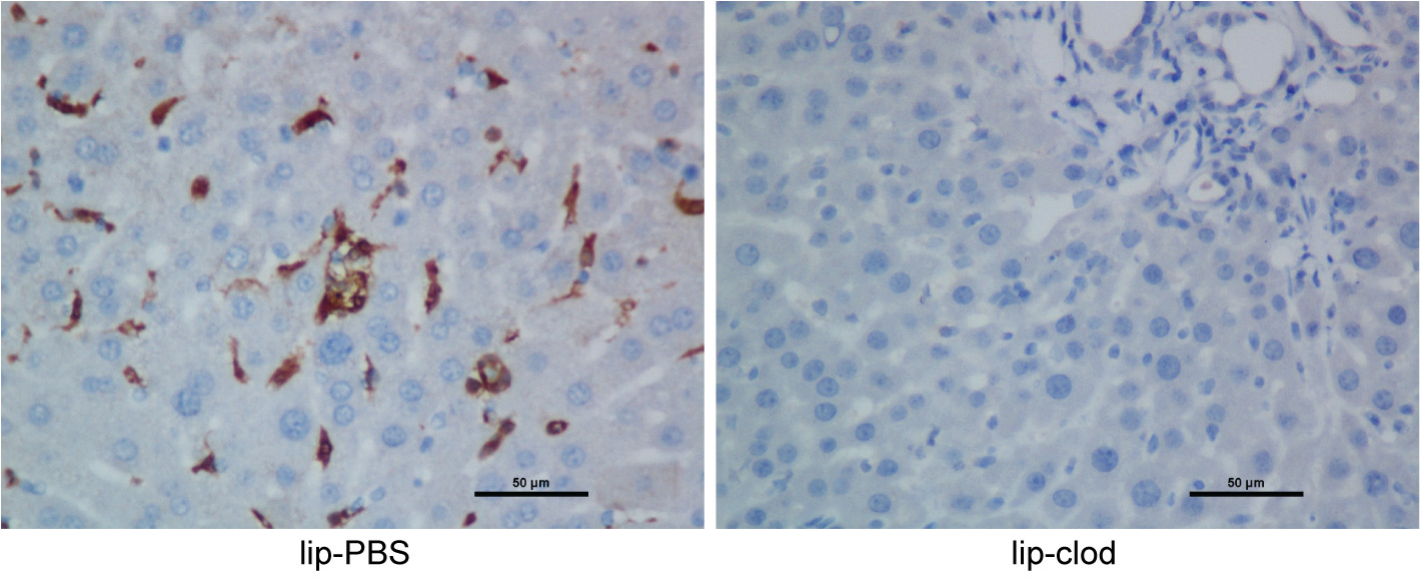
Kupffer cells were effectively eliminated in mouse livers after treatment with clodronate-containing liposomes. Immunohistochemical stain of livers from control mice treated with PBS-containing liposomes (lip-PBS) and mice treated with clodronate-containing liposomes (lip-clod) using antibody against F4/80 (Original magnifications, 400×magnification).

**Fig 5:**
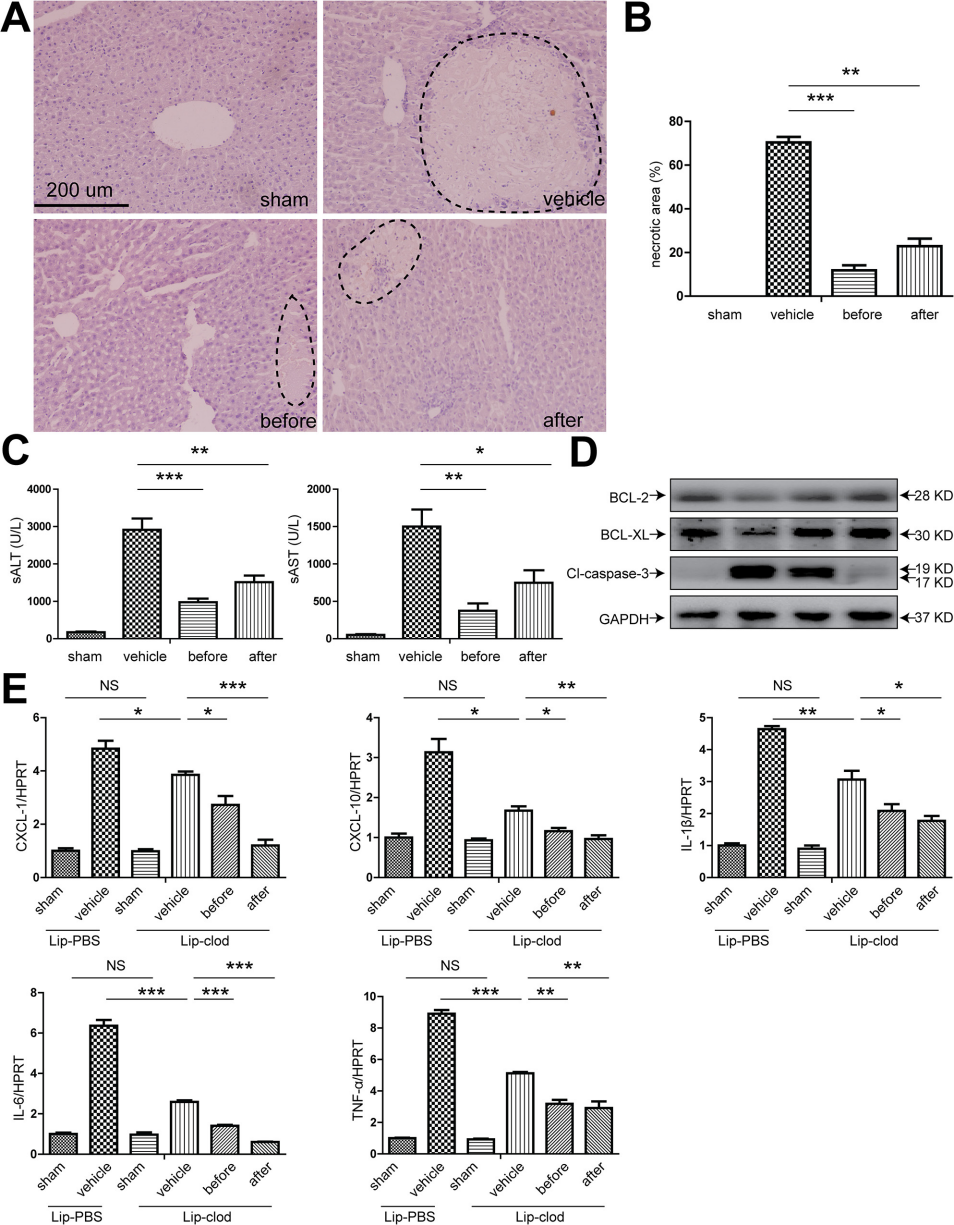
XBJ treatment ameliorated hepatic injury in lip-clod-treated mice. Lip-clod-treated B6 mice were either sham operated (sham group) or subjected to 90 minutes of partial warm ischemia, followed by 6 hours of reperfusion. XBJ (2 ml/kg iv) was given either before ischemia (before group) or after initiation of reperfusion (after group), while the same volume of normal saline was administered in the vehicle group. Serum and liver samples were harvested (n = 3-6/group). Hepatocellular function was analyzed by (A and B) liver histology (representative H&E staining; Original magnifications, 200×magnification) and (C) serum ALT and AST levels. (D) Western blot analysis of BCL-2, BCL-XL and cleaved-caspase-3 in IRI liver lobes. Representative blots from at least three mice for each time point were shown. (E) qRT-PCR detection of CXCL-1, CXCL-10, IL-1β, IL-6 and TNF-a. Data were normalized to HPRT gene expression and expressed as fold increase above the sham group of lip-PBS-treated mice (set as 1). Data were expressed as mean±SEM. *P<0.05, **P<0.01,*** P <0.001. NS, no significance.

**Fig 6:**
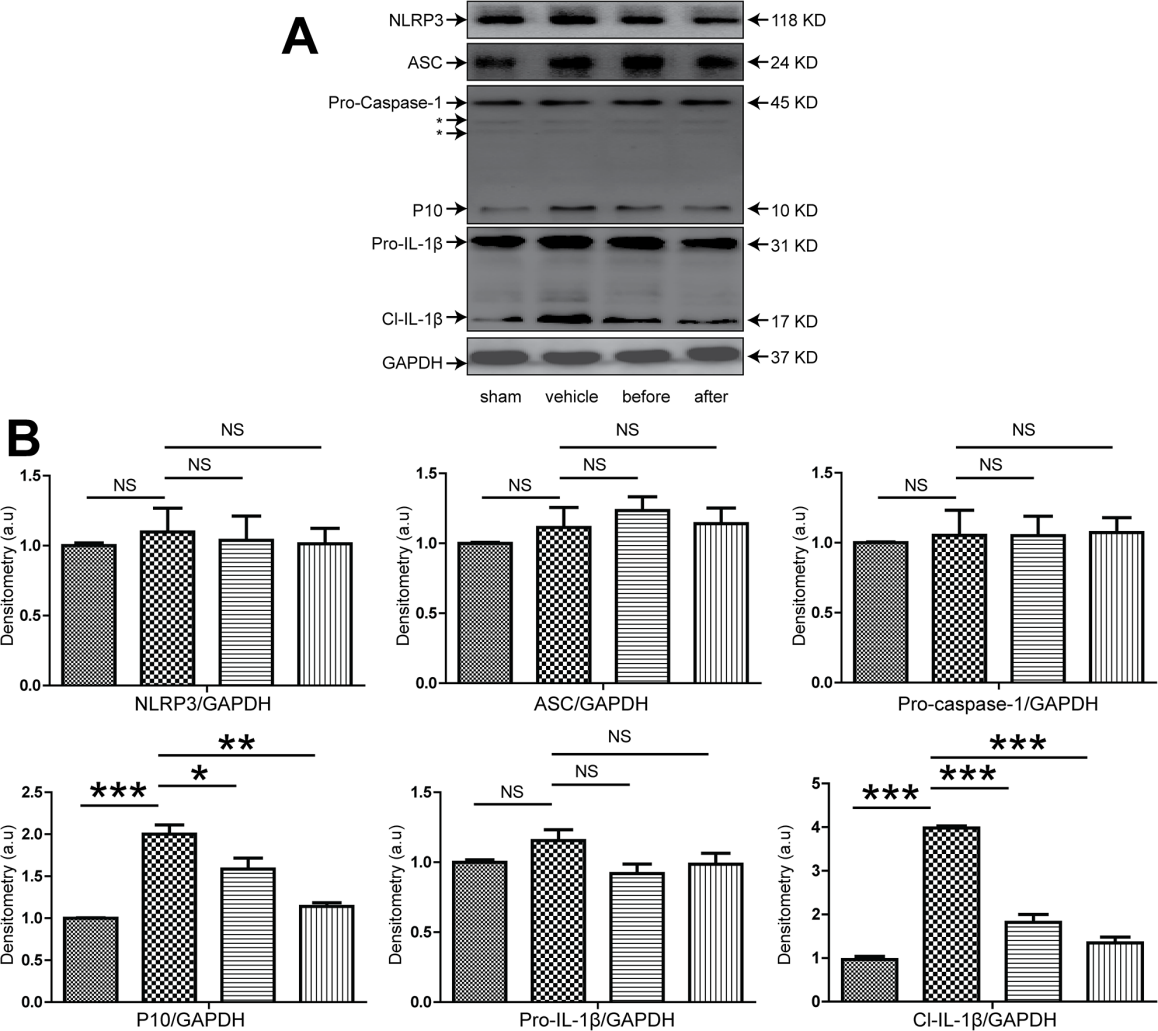
XBJ suppressed inflammasome activation in lip-clod-treated mice that have undergone IRI. Lip-clod-treated B6 mice were either sham operated (sham group) or subjected to 90 minutes of partial warm ischemia, followed by 6 hours of reperfusion. XBJ (2 ml/kg iv) was given either before ischemia (before group) or after initiation of reperfusion (after group), while the same volume of normal saline was administered in the vehicle group. Liver samples were then harvested (n = 3-6/group). Western blot analysis of NLRP3, ASC, Caspase-1 and IL-1β in IRI liver lobes. Representative blots from at least three mice were shown. *, non-specific bands. (B) Densitometric analyses of western blot for NLRP3, ASC, Caspase-1 and IL-1β. Values shown were relative to sham group (set as 1). Compared to sham group, *p<0.05; **p<0.01; ***p<0.001. NS, no significance.

Previous studies revealed that both hepatocytes and liver resident macrophage Kupffer cells played major roles in liver IRI, however the relative contribution of individual cell type in this pathological condition remains unclear. We used a commonly exploited method to deplete kupffer cells in vivo using lip-clod. After the mice were treated with lip-clod, immunohistochemical staining of the liver using antibody against F4/80 clearly showed a near-complete extinction of F4/80^+^ cells within the liver, suggestive of effective elimination of kupffer cells in vivo (Fig 4). Then these mice were either sham-operated or subjected to 90 mins partial warm ischemia followed by 6 hours of reperfusion with or without XBJ treatment. We found that in absence of kupffer cells in vivo, XBJ treatment still demonstrated potent effect in protecting liver from IRI as shown by liver pathology and biochemistry (Fig 5A-5C). Surprisingly, we found that the presence or absence of kupffer cells didn’t seem to greatly impact the extent of liver damage, given the similar magnitude of liver function tests and lobetic necrosis, suggesting that hepatocytes may play a more critical role in the pathological process of IRI. Similar to what have been done in WT mice, we also checked the expression of apoptosis related proteins in the liver. In mice with kupffer cell depletion, XBJ treatment still resulted in decreased Caspase 3 cleavage as well as increased expression of Bcl-2 and Bcl-XL in IR lobes (Fig 5D). Next, we looked at the generation of inflammatory cytokines/chemokines within the IR liver tissues using real-time PCR. Though lip-clod treatment had no significant effects on the gene expression of IL-1β, IL-6, TNF-α, CXCL1 and CXCL10 in sham-operated groups, the expression of these gene were reduced after IRI (vehicle group) from lipo-clod-treated mice. Furthermore, we clearly found that XBJ treatment in these kupffer cell depleted mice had even less IL-1β, IL-6, TNF-α, CXCL1 and CXCL10 expression within ischemic livers in comparison to the vehicle group (Fig 5E). Lastly, we checked the hepatic expression of inflammasome related proteins. Although XBJ treatment marginally decreases NLRP3 expression after kupffer cell depletion, it did not change the expression of ASC. However, the cleavage of pro-Caspase 1 and pro-IL-1β were both decreased substantially as compared to the vehicle treated group, suggesting that the intra-hepatic inflammasome activation was also greatly inhibited by XBJ use in the absence of Kupffer cells (Fig 6).

### 4. XBJ effectively ameliorated Redox-induced injury and inflammasome activation in isolated hepatocytes

**Fig 7:**
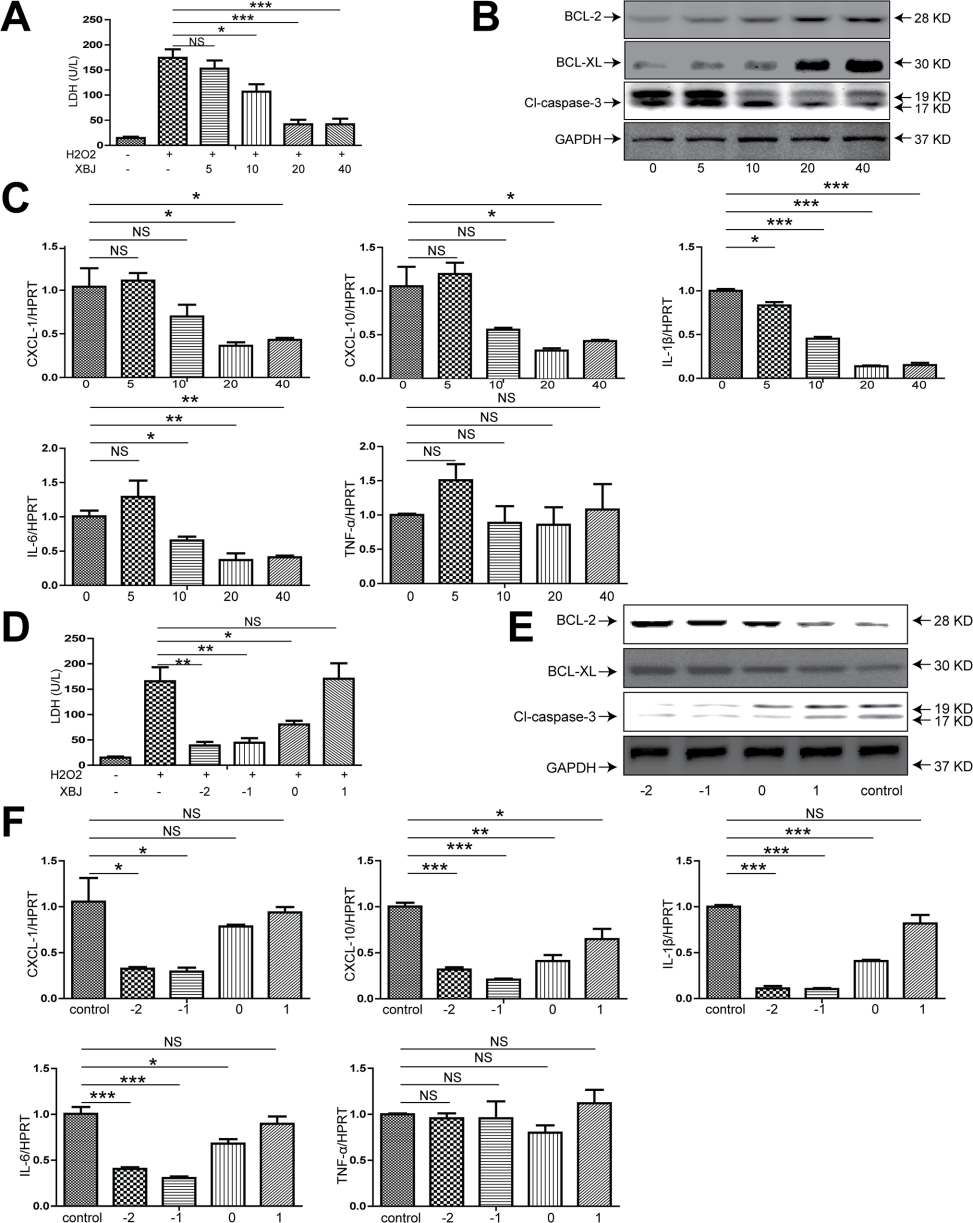
XBJ prevented H_2_O_2_-induced hepatocyte injury. (A-C) Hepatocytes were treated with increasing concentrations of XBJ (5, 10, 20 and 40 μl/ml) for 1 hour, and then subjected to H_**2**_O_**2**_ (200μM) for 5 hours. (A) LDH levels in the supernatant of cultured hepatocytes. (B) Cell lysates were analyzed for the protein expression of BCL-2, BCL-XL and cleaved caspase-3 by Western blot. (C) qRT-PCR detection of CXCL-1, CXCL-10, IL-1β, IL-6 and TNF-α in hepatocytes. Data were normalized to HPRT gene expression and expressed as fold increase above the H_**2**_O_**2**_ 200uM and 0 μl/ml XBJ treatment group (set as 1). (D-E) Hepatocytes were treated with H_**2**_O_**2**_ (200uM) for 5 hours. XBJ (20ul/ml) were added before H_**2**_O_**2**_ treatment for 2,1 or 0 hours or after H2O2 treatment for 1 hour. (D) LDH levels in the supernatant of cultured hepatocytes. (E) Cell lysates were analyzed for the protein expression of BCL-2, BCL-XL and cleaved caspase-3 by Western blot. (F) qRT-PCR detection of CXCL-1, CXCL-10, IL-1β, IL-6 and TNF-a in hepatocytes. Data were normalized to HPRT gene expression and expressed as fold increase above the H_**2**_O_**2**_ 200uM and 0 μl/ml XBJ treatment group (control group, set as 1). Data were expressed as mean±SEM.*P<0.05, **P<0.01,*** P <0.001, n = 3-6/group. NS, no significance.

**Fig 8:**
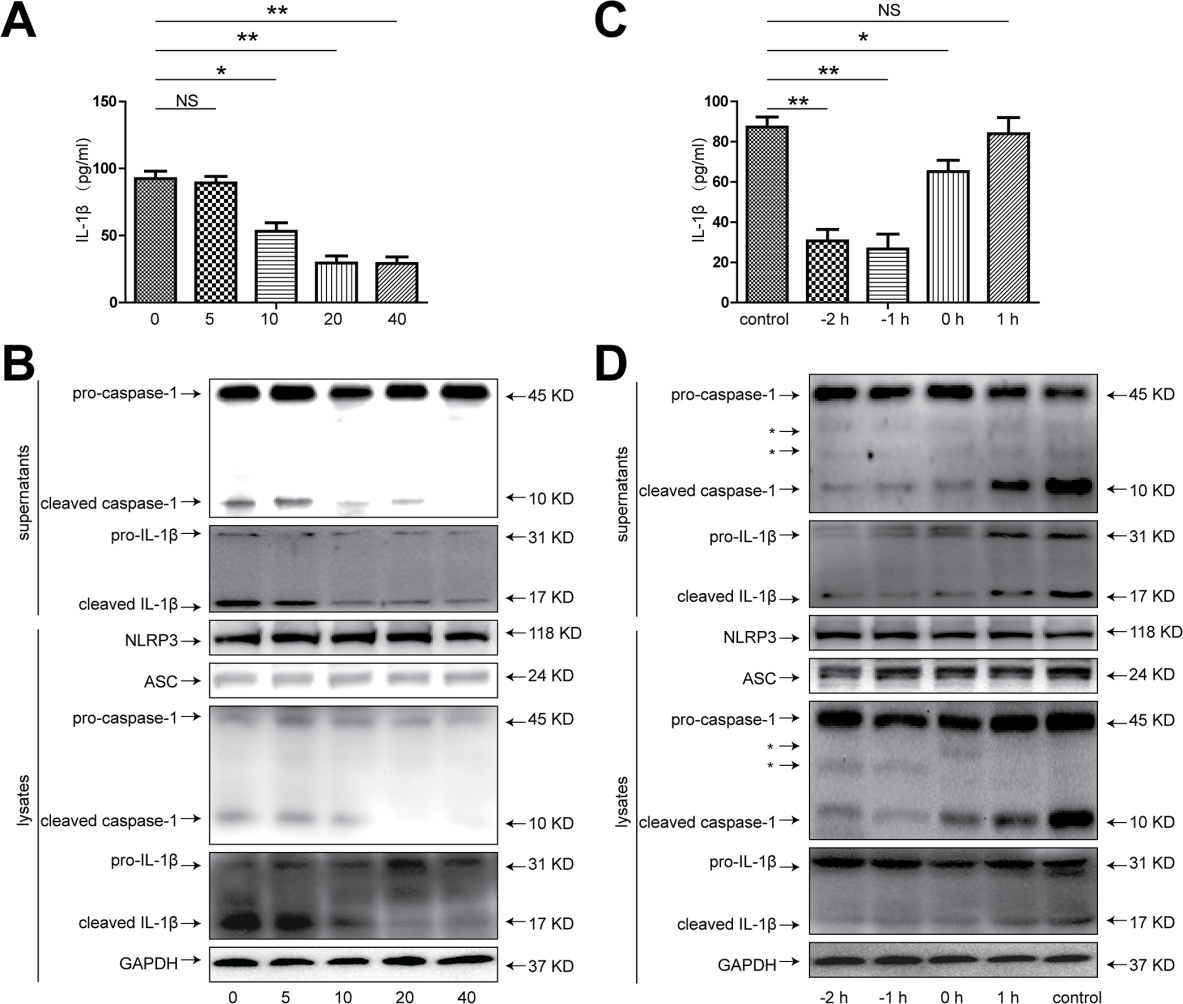
Time- and dose-dependent suppression of activation of inflammasome by XBJ in H_2_O_2_-treated hepatocytes. (A and B) Hepatocytes were treated with increasing concentrations of XBJ (5, 10, 20 and 40 μl/ml) for 1 hour, and then were treated with H_**2**_O_**2**_ (200uM) for 5 hours. (A) ELISA detection of the IL-1β protein levels in the culture medium. (B) Samples of cell lysate and culture medium were analyzed for the protein expression of NLRP3, caspase-1 and IL-1β by western blot. (C and D) Hepatocytes were treated with H_**2**_O_**2**_ (200uM) for 5 hours. XBJ (20ul/ml) was added before H_**2**_O_**2**_ treatment for 2, 1 or 0 hours or after H_**2**_O_**2**_ treatment for 1 hour. (C) ELISA detection of the IL-1β levels in the culture medium. (D) Samples of cell lysate and culture medium were analyzed for the protein expression of NLRP3, caspase-1 and IL-1β by Western blot. Data were expressed as mean±SEM.*P<0.05, **P<0.01, n = 3-6/group. NS, no significance. *, non-specific bands.

**Fig 9:**
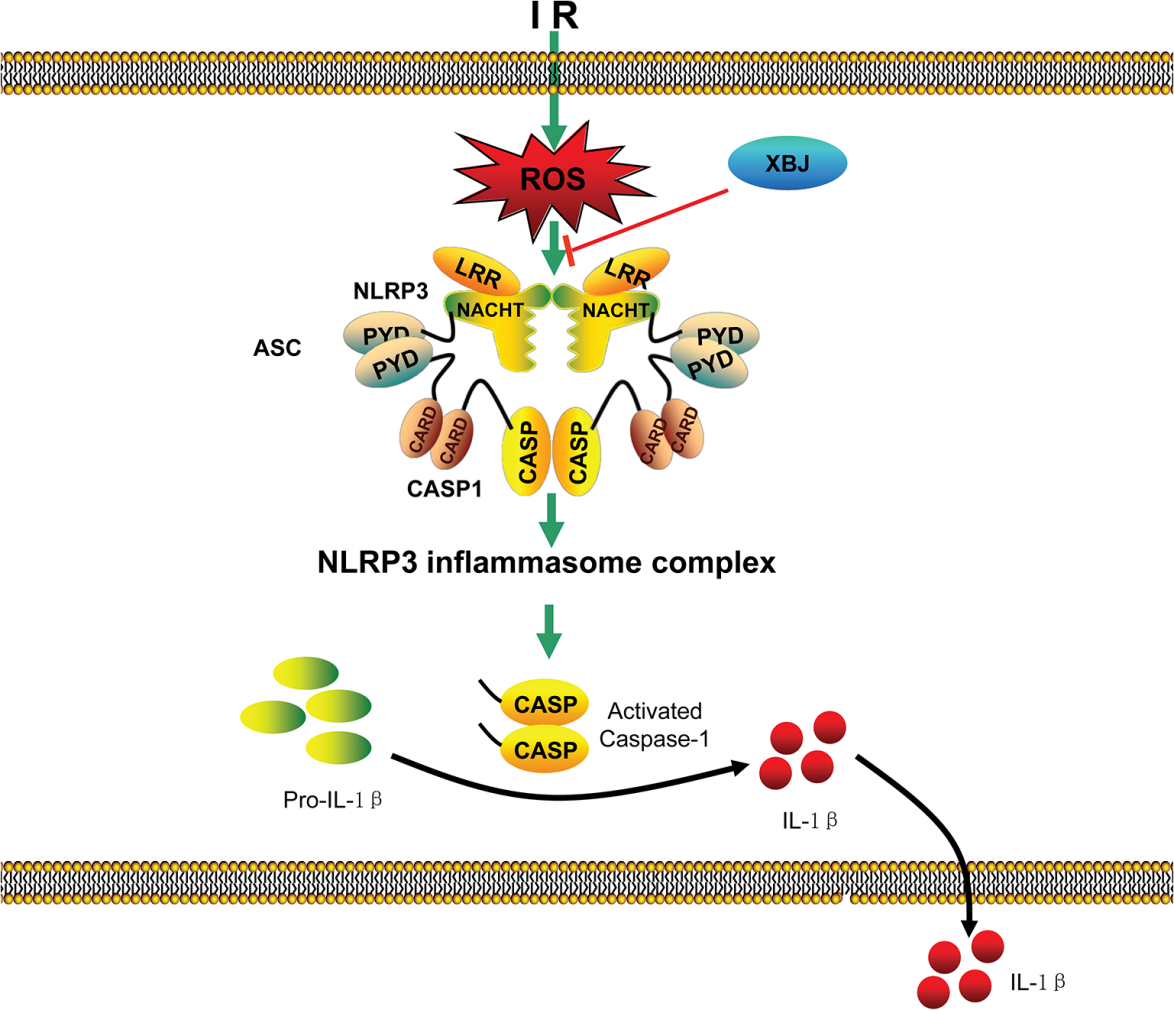
Schematic diagram of the proposed role of XBJ in hepatic IR. XBJ mainly blocked the activation of inflammasome.

Although the main ingredient of XBJ, HSYA, has been previously reported to lessen the rodent liver IRI which might be attributed to its suppressive effect on the secretion of inflammatory cytokines by Kupffer cells[31], our data with Kupffer cell depletion clearly pointed to the previously ignored fact that hepatocytes per se might be the main target for XBJ to exert it’s anti-apoptotic and anti-inflammatory effects. To further prove that XBJ has a direct effect on hepatocytes, we established an in vitro system using isolated primary hepatocytes. These cultured mouse hepatocytes were subjected to redox-induced injury using H_2_O_2_. Hepatocytes were treated with escalating concentrations of XBJ for 1 hour and then were subjected to H_2_O_2_ for 5 hours. We noticed a dose-dependent decrease of LDH levels in the supernatant, indicating less hepatocyte injury after XBJ treatment (Fig 7A). XBJ treatment also significantly decreased Caspase-3 cleavage as well as increased Bcl-2 and Bcl-XL expression in hepatocytes subjected to H_2_O_2_-induced injury in a dose dependent manner (Fig 7B). Next we looked at the hepatocyte production of a similar panel of inflammatory cytokines/chemokines after H_2_O_2_ treatment in vitro in the presence and absence of XBJ. Clearly, we also identified a dose-dependent decrease of the production of CXCL1, CXCL10, IL-1β, IL-6 but no TNF-α in isolated hepatocytes (Fig 7C). In the next part of the experiment, we tried different time points of XBJ treatment in those hepatocytes subjected to H_2_O_2_ treatment. We either pre-treated these hepatocytes with XBJ at various time points prior to or after H_2_O_2_ mediated injury, and identified XBJ was capable of significantly ameliorating hepatocyte injury only before but not after H_2_O_2_ treatment (Fig 7D). This was associated with less hepatocyte apoptosis as shown by decreased Caspase-3 cleavage, increased expression of anti-apoptotic Bcl-2 and Bcl-XL as well as decreased production of inflammatory cytokines/chemokines including CXCL1, CXCL10, IL-1β, IL-6 but not TNF-α (Fig 7E and 7F). Lastly, we examined whether XBJ had a direct impact on inflammasome activation in H_2_O_2_-treated hepatocytes. IL-1β secretion was inhibited in a dose dependent manner after XBJ treatment (Fig 8A). Furthermore, Using western blot to check the inflammasome related protein expression in either the culture supernatant or the hepatocytes per se, we found that XBJ treatment significantly decreased the H_2_O_2_ induced Caspase-1 as well as IL-1β cleavage, while the NLRP3 and ASC expression were not significantly changed, indicating the effective inhibition of hepatocyte inflammasome activation after XBJ treatment in a dose-dependent manner (Fig 8B). Finally, consistent with the time-dependent protective effect of XBJ on hepatocyte injury, IL-1β secretion and the inflammasome activation of hepatocytes was also dramatically inhibited by XBJ in XBJ pre-treated hepatocytes but not after these hepatocytes were subjected to H_2_O_2_ treatment (Fig 8C and 8D). These data further supported a direct modulatory effect of XBJ on hepatocyte inflammasome activation and apoptosis in a time- and dose-dependent manner (Fig 9).

### 5. Systematic study of XBJ’s anti-inflammatory effects using network pharmacology technology

**Fig 10:**
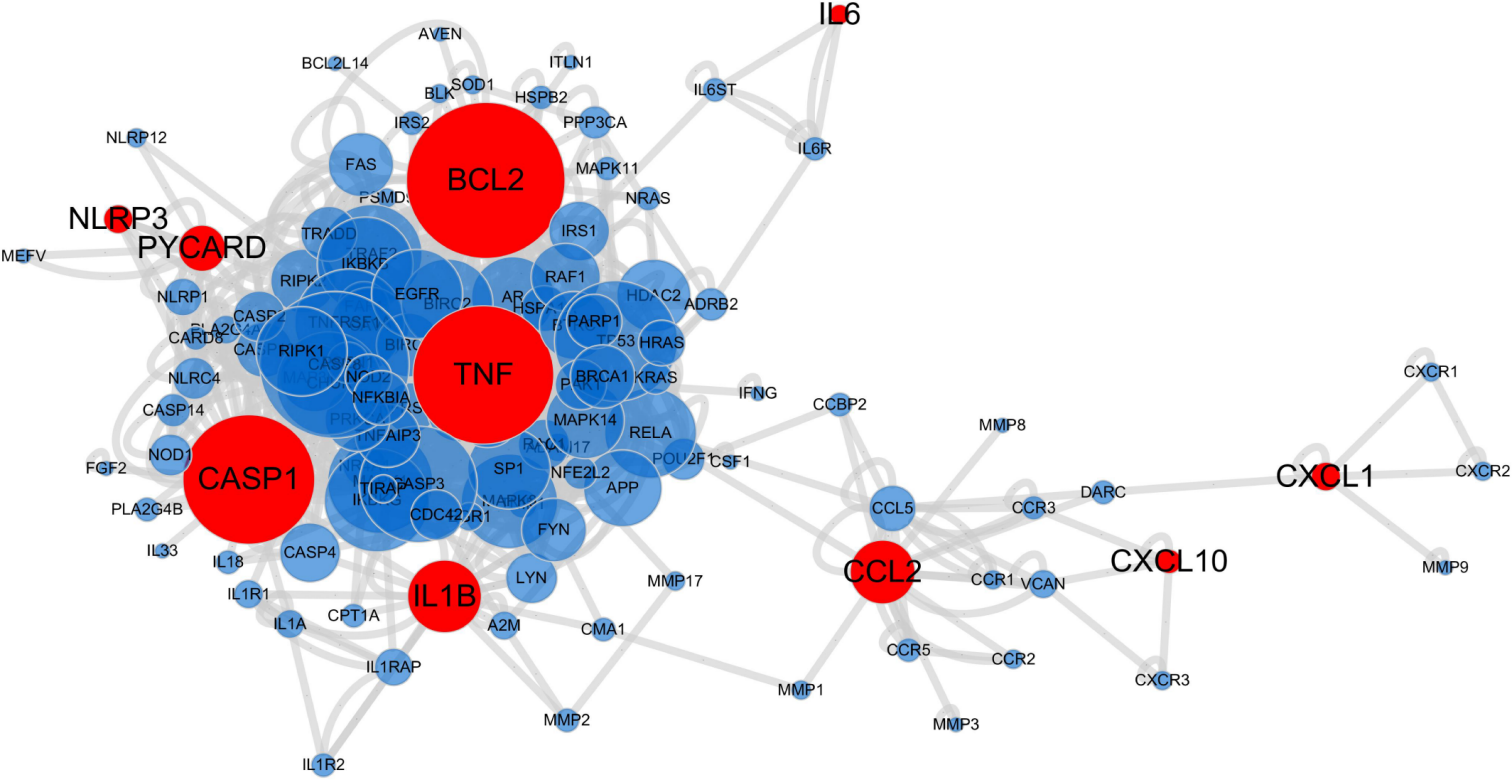
Multi-target anti-inflammatory effect of XBJ Injection. The inflammation associated network was constructed using network pharmacology technology, in which XBJ showed anti-inflammatory effect through a multi-target mode of action. This network consisted of nodes and edges, and inflammation associated nodes were linked through edges, while red nodes stand for targets of XBJ with experimental investigations at different levels (*i*.*e*. BCL2, CASP1, TNF, IL1B, CCL2, PYCARD, NLRP3, CXCL1, CXCL10, IL6). Size of node was in proportion to the numbers of links to the node, that is the bigger size node had more associated genes. Inflammation associated genes were obtained from HuGE Navigator (version 2.0)

Although the traditional Chinese medicine (TCM) has long been recognized as alternative or complementary to western medicine and TCM shows good clinical effectiveness especially for some complex diseases and disorders, the molecular mechanisms of many TCM formula are not systematically understood, including XBJ. Network pharmacology is the next paradigm in drug discovery and illuminates our understanding of complex drug-target interactions[40,41], so it is especially suitable for many traditional Chinese herbal preparations with multiple drug targets. In our study, network pharmacology technology was applied to integrate our experimental results. HuGE Navigator is a continuously updated knowledge base in human genome epidemiology, including detailed information about gene-disease associations, so the inflammation related genes were collected and used for construction of the gene interaction network [36]. We found this method provided a visualization way to show XBJ has anti-inflammatory effects through a multi-target, multi-pathway mode of action at a molecular network level. The network (Fig 10) indicated the potential targets of anti-inflammation effect, which could also be the targets of XBJ. As shown in Fig 10, some targets of XBJ were highlighted in red and were investigated experimentally in different levels in our study, while other potential targets of XBJ need further investigations. Nowadays, network pharmacology and network analysis are accepted as important methods to systematically dissect the complex mode of actions of TCM for complex diseases treatment. Combined with-omics technologies, network pharmacology can be applied to discover targets/new targets and active components of TCM, which could facilitate the development of TCM with more effectiveness but less side effects.

## Discussions

In the current study, we used both in vivo IRI model and in vitro H_2_O_2_-induced hepatocyte injury model to study the effect of XBJ on hepatic function, hepatocyte apoptosis and inflammasome activation. We proved that XBJ administration could become a simple and effective means to minimize liver IRI in mouse models. In particular, we provided the first line of evidence that in addition to their proposed inhibitory effects on kupffer cell activation, XBJ had potent anti-apoptotic and anti-inflammatory effects on hepatocytes in a time- and dose-dependent manner. More importantly, besides its several known components to inhibit NF-κB activity, this is the first report which clearly demonstrated that XBJ interfered with the proper function of the inflammasome protein complex. Given the unchanged expression levels of NLRP3 and ASC proteins, we further hypothesized that XBJ affected the assembly or function of NLRP3 inflammasome, rather than the protein expression of inflammasome components.

IR-mediated hepatic damage consists of acute injury resulting from hypoxic cellular stress and inflammation-mediated reperfusion injury. The regenerative process after hepatic IRI involves the clearance of dead tissue and the hepatocyte proliferation[42]. Kupffer cells reside within the liver sinusoid and serve as the first line of defense to injury. They produce pro- and anti-inflammatory cytokines and other biologically important molecules upon engagement of the pattern recognition receptors such as Toll-like and nucleotide-binding oligomerization domain (NOD)-like receptors. Kupffer cells show either pathological or beneficial actions in the context of varying pathological processes via the activation of different machineries they possess[43]. Kupffer cells are the one of important producer of ROS and contribute to the early phase of IRI. In addition, their production of proinflammatory cytokines propagates the inflammatory response throughout the liver and induces the hepatic expression of CXC chemokines and endothelial adhesion molecules[44]. We originally hypothesized that XBJ suppressed the release of proinflammatory cytokines of Kupffer cells and reduced the hepatic injury. To our surprise, treatment with XBJ ameliorated hepatic IRI to a comparable magnitude in livers with selective depletion of Kupffer cells. Although depletion of Kupffer cells partially decreased liver production of pro-inflammatory cytokines/chemokines as expected (Fig 5E), it rarely affected hepatocyte apoptosis (Fig 5D), suggesting hepatocytes per se may play a dominant role in liver IRI and may be the major target of XBJ therapy. These results prompted us to examine the effect and mechanism of XBJ on hepatocytes directly.

The NLRP3 infammasome is a protein complex that stimulates Caspase-1 activation to promote the processing and secretion of proinfammatory cytokines, such as IL-1β and IL-18 [45]. Both NLRP3 and its downstream target caspase-1 were activated during IR and were essential for hepatic IRI [46]. NLRP3 inflammasome activation in hepatocytes and nonparenchymal cells resulted in induction of proinflammatory signaling and hepatocyte apoptosis[47]. The activation of caspase-1/IL-1β pathway promoted high mobility group box-1 (HMGB1) protein induction to facilitate a TLR4-dependent inflammatory phenotype leading to IR hepatocellular damage [48]. Gene silencing of NLRP3 results in protection from inflammation and hepatocyte injury after liver IRI. This protective effect is associated with reduced production of proinflammatory cytokines, including IL-1β, IL-18, TNF-α, and IL-6 [46]. NLRP3 knockout would also protect against ischemic acute kidney injury[49].

IL-1β is an important cytokine that induces inflammatory injury during hepatic IR. Previous study reported that IL-1β could stimulate NF-κB activation, hepatic neutrophil accumulation, and the production of pro-inflammatory cytokines[48,50]. Moreover, IL-1β could induce up-regulating of ROS after liver IRI. Inhibition of endogenous IL-1β activation protected livers against IRI[51]. However, there is no evidence that IL-1β directly caused hepatocyte apoptosis[52]. Nevertheless, some studies even showed that IL-1β could protect mice from liver injury and attenuate hepatocyte apoptosis[52,53]. This contradictory effect need to be elucidated in future studies.

XBJ is a compound preparation made from traditional Chinese medicines. Partial least square discrimination analysis (PLS-DA) was performed on chemical fingerprint data and twenty-one compounds were identified or tentatively characterized [54]. Multiple bioactive constituents, such as safflower yellow A, tetramethylpyrazine, danshensu and ferulic acid were identified, and these components were considered to be largely responsible for the therapeutic effects of XBJ [11]. However, we can’t find any literature regarding the in vivo pharmacokinetics of XBJ.

In our study, the hepatoprotective effect of XBJ after IR was mainly due to its anti-inflammatory and anti-apoptotic actions. Consistent with the previous study[12,15], our data confirmed that the anti-inflammatory effects was partly due to the inhibition of the mRNA and protein expression of the pro-inflammatory cytokines. More importantly, our data showed for the first time that the anti-inflammatory and anti-apoptotic properties of XBJ could both be attributed to the suppression of activation of NLRP3 inflammasome (Fig 9). Although further investigations are required, our results clearly indicate that XBJ is a simple and promising means for the treatment of liver IRI.

A caveat of the current study is we don’t know which component or components are responsible for its anti-apoptotic and anti-inflammatory effects of XBJ as well as the mechanism involved. Several components of XBJ have been identified to inhibit NF-κB-dependent inflammatory gene expression, including gallic acid, danshensu, hydroxysafflor yellow A, etc[9,22,55]. Moreover, it is critical to further dig into the mechanism of how XBJ and its active component interfere with proper inflammasome function. Besides, we don’t know whether other inflammasomes such as the absent in melanoma 2 (AIM2) may also be target of XBJ in addition to NLRP3. Finally, although XBJ has been reported to exert anti-apoptotic function in many cell types[9,22,55], the mechanism of anti-apoptosis in hepatocytes remains to be elucidated. These interesting questions are beyond the scope of the current study and needed to be addressed in future investigations. Nevertheless, our novel and important finding regarding the direct effect of XBJ on inflammasome activation will definitely prompt us to further screen out the molecules in XBJ preparation which are most potent in the inhibition of inflammasome activation, which has important implications in inflammasome-targeted drug discovery.

In summary, we find that the Chinese herbal preparation XBJ has potent protective effect against the liver IRI in mice, mainly due to its anti-inflammatory and anti-apoptotic effects. Besides its known effect on Kupffer cell production of inflammatory cytokines/chemokines through a NF-κB-dependent mechanism, we find the amelioration of hepatic IRI by XBJ can be largely attributed to its direct effect on hepatocyte inflammasome activation and Caspase 1-dependent IL-1β production. Our findings not only provide mechanistic insights into the anti-inflammatory effect of XBJ, but can be easily translated into a simple and effective therapeutic means in many clinical scenarios given its routine use in the management of many septic disorders.
